# Mediator role of physical activity in the relationship between anthropometric indicators and blood pressure in children with normal weight and children with overweight/obesity

**DOI:** 10.1186/s12889-025-25997-8

**Published:** 2026-03-11

**Authors:** Maiara Cristina Tadiotto, Neiva Leite, Caroline Brand, Ana Duarte, Claudia Augusto, Maria José Silva, Jorge Mota, Beatriz Pereira, Rafaela Rosário

**Affiliations:** 1 Research Nucleus On the Quality of Life, NQV, Federal University of Paraná, Curitiba, Brazil; 2 Research Centre On Child Studies, CIEC, University of Minho, Braga, Portugal; 3 Research Centre for Physical Activity, Health and Leisure, CIAFEL, University of Porto, Porto, Portugal; 4 IRyS Group, Physical Education School, Pontificia Universidad Católica de Valparaíso, Valparaíso, Chile; 5 School of Nursing, University of Minho, Braga, Portugal; 6 Health Sciences Research Unit: Nursing (UICISA: E), Nursing School of Coimbra, Coimbra, Portugal

**Keywords:** Adiposity, Children, Blood pressure, Hypertension, Physical activity

## Abstract

Obesity is associated with comorbidities, including hypertension, which have been diagnosed in the pediatric population. The aim of this study was to verify the mediator role of physical activity in the relationship between anthropometric indicators and blood pressure (BP) in children with normal weight and children with overweight/obesity. The BeE-school project included 728 Portuguese children (376 boys, 352 girls), aged 6 to 10 years old, who were divided into two groups: children with normal weight and children with overweight/obesity, according to body mass index z-score (BMI-z). Body mass, height, waist circumference (WC), systolic BP (SBP) and diastolic BP (DBP) were assessed. Tri-ponderal mass index (TMI), waist-to-height ratio (WHtR) and mean BP (MBP) were calculated. Sedentary behavior and moderate-to-vigorous physical activity (MVPA) were determined by accelerometry. Mediation analyses were conducted considering predictors (WC, WHtR, TMI), mediators (MVPA on weekdays, weekend, total), outcomes (SBP, DBP, MBP). Children with overweight/obesity had higher mean values for BP measurements compared to their normal weight peers. In the correlations were not observed associations between anthropometric indicators and BP variables in children with normal weight, but MVPA on weekdays and MVPA total showed positive associations with SBP. However, children with overweight/obesity presented significant positive associations between all anthropometric indicators and BP variables. The present study provides evidence that physical activity functions as a competitive mediator in relationship between TMI and BP in children with overweight/obesity. The overweight simultaneously elevates BP directly while reducing protective physical activity engagement in Portuguese children.

## Contribution to Health Promotion

- Demonstrates that excessive adiposity in childhood contributes to early increases in blood pressure and cardiovascular risk.
- Highlights the protective role of regular moderate-to-vigorous physical activity in maintaining healthy blood pressure levels.
- Reveals that children with overweight or obesity exhibit reduced physical activity engagement, compromising cardiovascular regulation.
- Identifies the tri-ponderal mass index (TMI) as a sensitive anthropometric indicator for detecting obesity-related cardiometabolic risk.
- Supports integrated health-promotion strategies in schools that combine weight management and structured physical activity to prevent hypertension from early life.

## Introduction

Promoting health and preventing chronic non-communicable diseases should begin in childhood and adolescence. Early identification of hypertensive measures is essential [5], as their frequency has increased in several countries in association with obesity, including in Portuguese children and adolescents [30]. Childhood obesity constitutes one of the main threats to global public health. It is estimated that more than 35 million children under 5 years of age are overweight, and globally, more than 390 million individuals aged 5–19 years live with overweight [39]. In Portugal, overweight is estimated at 25% in children under 10 and 32% in adolescents aged 10 to 17 [17], because of increased sedentary behavior, inadequate diet and a reduction in regular physical activity [7], factors amplified by the isolation of the coronavirus pandemic [14].

In cross-sectional studies, the prediction of blood pressure (BP) changes in the pediatric population can be predicted in a similar way by body mass index (BMI) or waist circumference (WC) [22]. Although BMI is traditionally used to estimate cardiovascular risk, recent studies have proposed evaluating BP using the tri-ponderal mass index (TMI) [12, 15], which showed that it can better discriminate body fat distribution than BMI [20]. Despite this, there is still controversy regarding which anthropometric indicator best predicts BP in prepubertal ages, as results are heterogeneous.

These results demonstrate the importance of epidemiological studies and monitoring of anthropometric measurements and BP in schoolchildren [24], as a method of prevention and health surveillance of the child and adolescent population. They also reinforce the existence of gaps in relation to the most appropriate anthropometric predictor of increased BP, especially considering that this relationship has been little explored in children under 10 years of age.

Regarding the practice of physical activities, studies have highlighted the favorable effect of aerobic physical exercise programs in reducing BP [35] and in hypertensive adolescents with obesity [16]. Regular physical activity in a school environment also contributes to a reduction in DBP [36], especially in children with overweight [38], while children in the hypertensive control group with obesity, there is a tendency to reduce systolic BP (SBP) during monitoring [16].

Thus, it can be observed that chronic non-communicable diseases risk factors are directly associated with obesity, while physical fitness is indirectly associated with these factors, since it contributes to reducing obesity levels and improving metabolic health in response to physical training [34]. However, evidence is still insufficient to confirm whether physical activity has an isolated mediating role in the association between adiposity and BP. In addition, there is a gap regarding the most appropriate anthropometric indicator for predicting BP changes in childhood. Therefore, the aim of this study was to verify the mediator role of physical activity in the relationship between anthropometric indicators and BP in children with normal weight and children with overweight/obesity.

## Materials and methods

### Study design and ethics

This is an observation cross-sectional study that is part of the BeE-school Project, which was conducted with schoolchildren in primary schools located in economically and socially disadvantaged areas characterized by poverty and social exclusion from the city of Braga, Portugal [21]. This research project was approved by the Ethics Subcommittee for Life and Health Sciences (CE.CVS 009/2022) at the University of Minho, in accordance with the Declaration of Helsinki. All parents and/or guardians were informed and signed an informed consent form, and the children were asked to assent to participate before the procedures. Data collection occurred from October to December 2022.

### Sample selection and participants

The sample selection process was conducted in ten primary schools. The inclusion criteria were applied: (a) children from primary schools prone to vulnerability; (b) age between 6 and 10 years; (c) no contraindications to carrying out assessments and tests. The exclusion criteria were: (a) non-participation in all the assessments; (b) children with impairments, whether cognitive or physical, that could compromise data collection. The sample consisted of 728 children (376 boys, 352 girls) between the ages of 6 and 10, divided into two categories according to body mass index score z (BMI-z): children with normal weight group and children with overweight/obesity group.

### Somatic maturation

Somatic maturation was determined by distance from the peak height velocity (PHV), using a mathematical model based on height, age, and sex. The prediction of age at PHV was determined by subtracting from the maturity-offset from the chronological age [23].

### Anthropometrics indicators

Body mass and height measurements were recorded using a pediatric scale/stadiometer (SECA 799 and SECA 224, respectively). Two measurements were taken by trained researchers using standardized procedures, with the children lightly dressed and without shoes. The procedure was repeated in case of doubt or uncertainty about the measurement. The body mass index (BMI) was calculated by the ratio of body mass to height squared (kg∙m^−2^) and converted into z scores (BMI-z) adjusted for specific age and sex categories [4]. BMI-z was categorized into two groups: children with normal weight (score ≤ 1), and children with overweight/obesity (score > 1).

Tri-ponderal mass index (TMI) was calculated by the ratio of body mass by height cubed (kg∙m^−3^). Waist circumference (WC) was measured in centimeters using a flexible, inextensible anthropometric tape, just 1 cm above the iliac crest, parallel to the ground, with the individual standing, abdomen relaxed, arms alongside the body and feet together. The waist-to-height ratio (WHtR) was calculated as ratio WC to height [1].

### Blood pressure

Two BP measurements were taken after resting in a sitting position for 10 min. The SBP and DBP were measured on the right arm at heart level, using a previously calibrated digital sphygmomanometer, with the cuff size appropriate to the individual's arm circumference. In case of doubt or uncertainty about the measurement, the procedure was repeated. Mean BP (MBP) was calculated using the sum of one-third of the SBP and two-thirds of the DBP.

### Sedentary behavior and physical activity

The schoolchildren were instructed to wear the triaxial Actigraph wGT3X-BT accelerometer (Actigraph LLC, Pensacola, FL, USA) continuously for 24 h a day over a 7-day period on the dominant wrist, except during water-related activities such as bathing or swimming. The research team conducted on-site visits to schools to place accelerometer devices on the participants and guided them with detailed instructions and documentation sheets to record pertinent information regarding the children’s daily activities and sleep patterns from the preceding night, including instances of non-wear and specific bedtime and wake-up times. After a week, the research team returned to retrieve the accelerometers, download data, and prepare the data for analysis.

The data were accessed and analyzed by the specific Actigraph wGT3X-BT software (version 6.13.5, Actigraph LLC). Periods of sleep and non-use were automatically identified by the software's validated algorithms and excluded from the analyses. Data was excluded when accelerometer usage time was below 16 h per day, on a minimum of three weekdays and one weekend day. The periods of physical activities were classified according to the acceleration intensity thresholds [10], according to the Euclidean Norm Minus One (ENMO). Sedentary behavior was defined as intensity of physical activity with ENMO < 35.0 mg, light physical activity (LPA) as ranging from ENMO 35.0 to 200.0 mg, and moderate-to-vigorous physical activity (MVPA) as ENMO > 200.0 mg [10, 11]. The mean time of sedentary behavior was recorded as hours per day, and the time spent on LPA and MVPA was recorded as the means of minutes per week and on the weekend.

### Statistical analysis

Descriptive statistics are reported as means and standard deviations. Differences between children with normal weight and children with overweight/obesity were examined using independent two-tailed *t*-tests. Pearson correlation analyses were performed to explore the associations among anthropometric indicators, BP variables, and physical activity levels.

**Fig. 1:**
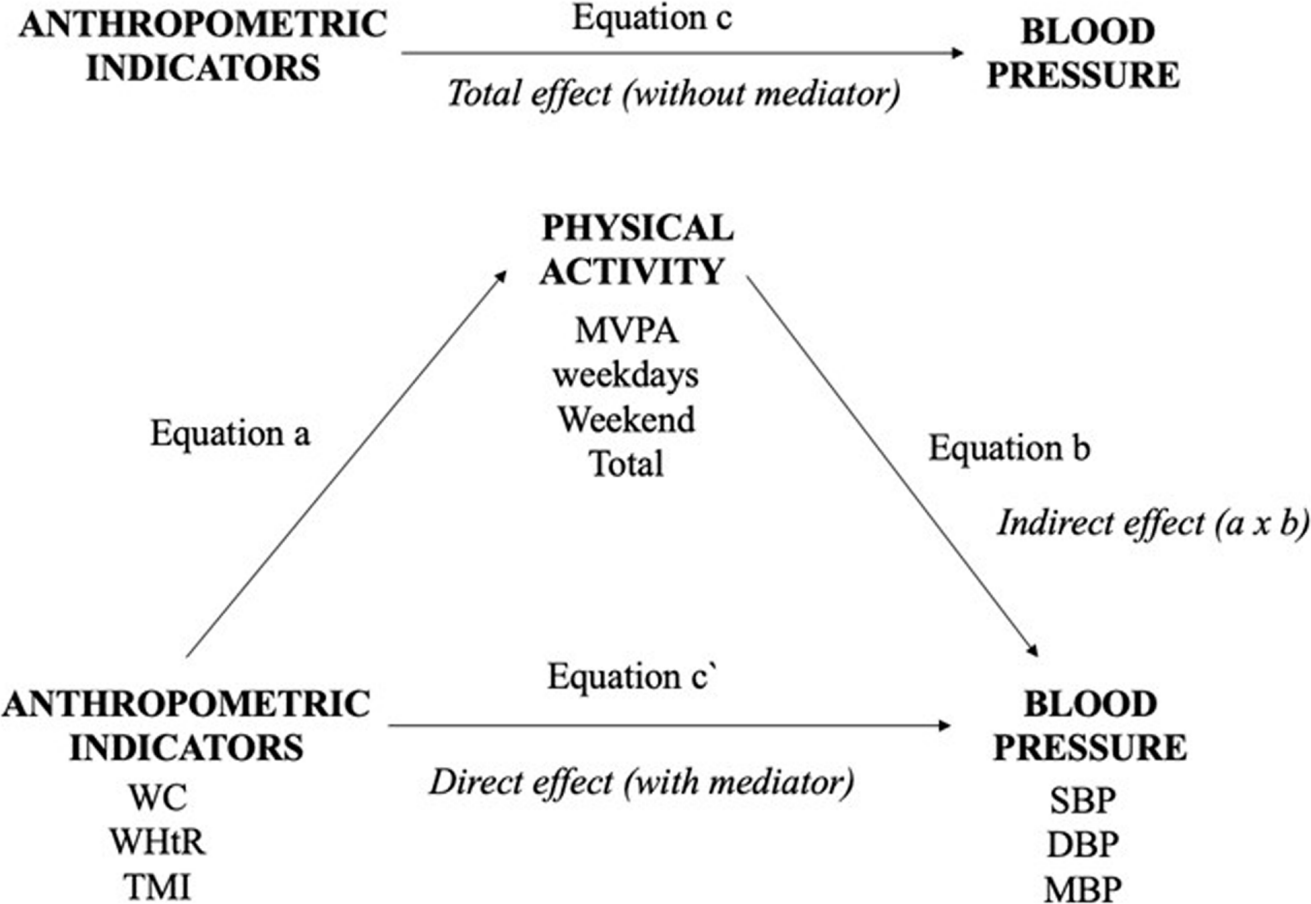
Theoretical approach of mediation model. MVPA. Moderate-to-vigorous physical activity; WC. Waist circumference; WHtR. Waist-to-height ratio; TMI. Tri-ponderal mass index; SBP. Systolic blood pressure; DBP. Diastolic blood pressure; MBP. Mean blood pressure

Mediation analyses were conducted considering predictors (WC, WHtR, TMI), mediators (MVPA on weekdays, weekend, and total), outcomes (SBP, DBP, MBP) (Fig. 1). The mediation analysis followed a structured approach involving four equations: (a) the association between the predictor (anthropometric indicators) and the mediator (MVPA); (b) the association between the mediator and the outcome (BP); (c) the total effect of the predictor on the outcome, without including the mediator; and (c′) the direct effect of the predictor on the outcome, adjusted for the mediator. To assess mediation effects, linear regression models with bootstrapping (5,000 resamples, 95% IC BCa) were conducted using the PROCESS macro for SPSS version 27.0 (IBM Corp., Armonk, NY, USA) [9], based on the approach proposed by Preacher and Hayes [29]. An indirect effect was considered statistically significant when the 95% confidence interval did not include zero [6].

Finally, mediation effects were classified according to the framework proposed by Nitzl et al. [25]. The categories were defined as follows: (a) Indirect-only (full mediation): a significant indirect effect is present, but no direct effect is observed,(b) Complementary (partial mediation): both indirect and direct effects are significant and point in the same direction, indicating that the mediator partially explains the effect of the predictor on the outcome; (c) Competitive (partial mediation): both effects are significant but in opposite directions, suggesting a suppression or compensatory mechanism; (d) Direct-only (no mediation): only the direct effect is significant, with no evidence of mediation; and (e) No effect—neither direct nor indirect effects are significant [41]. All models were adjusted for sex, PHV, height, and sedentary behavior. Statistical significance was set at p < 0.05 for all analyses.

## Results

Table 1 presents the descriptive characteristics of the total sample and stratified by body mass status. The data shows that children with overweight/obesity had higher mean values for all anthropometric indicators (TMI, WC, and WHtR) compared to their normal weight peers (p < 0.05). A similar trend was observed for BP measurements. Regarding physical activity, children with overweight/obesity engaged in lower levels of physical activity during weekdays, weekends, and across the entire week (p < 0.05). Additionally, they exhibited higher sedentary behavior during the weekend (p < 0.05).

**Table 1:** Descriptive characteristics of the total sample and by nutritional status (mean ± SD)

**Variables**	**Total** (*n* = 728)	**Normal weight** (*n* = 446)	**Overweight/obesity** (*n* = 282)
Age (years)	8.2 ± 1.2	8.1 ± 1.2	8.3 ± 1.2*
PHV (maturity offset)	−3.22 ± 0.9	−3.34 ± 0.9	−3.01 ± 1.0**
Height (cm)	130 ± 9.4	128 ± 8.7	134 ± 9.2**
Body mass (kg)	30.3 ± 8.8	25.8 ± 4.8	37.4 ± 9.2**
TMI (kg∙m^−3^)	13.5 ± 2.1	12.31 ± 1.0	15.4 ± 1.8**
WC (cm)	60.9 ± 8.8	55.9 ± 4.3	68.6 ± 8.4**
WHtR	0.46 ± 0.05	0.43 ± 0.02	0.51 ± 0.04**
SBP (mmHg)	103.0 ± 11.1	102.0 ± 10.8	104.8 ± 11.4**
DBP (mmHg)	62.2 ± 9.3	61.6 ± 9.1	63.2 ± 9.6*
MBP (mmHg)	75.8 ± 9.0	75.1 ± 8.7	77.0 ± 9.3*
**Sedentary behavior and physical activity (weekdays)**			
Sedentary behavior (h∙day)	8.7 ± 1.6	8.7 ± 1.6	8.8 ± 1.5
LPA (min∙day)	312.0 ± 50.6	311.0 ± 52.2	314.0 ± 47.8
MVPA (min∙day)	77.5 ± 31.2	79.7 ± 33.1	74.4 ± 27.7*
**Sedentary behavior and physical activity (weekend)**			
Sedentary behavior (h∙day)	9.3 ± 1.84	9.1 ± 1.96)	9.5 ± 1.6*
LPA (min∙day)	273.0 ± 65.0	277 ± 67.6	265.0 ± 60.1
MVPA (min∙day)	54.5 ± 29.8	56.9 ± 30.0	50.7 ± 29.3*
**Sedentary behavior and physical activity (total)**			
Sedentary behavior (h∙day)	8.9 ± 1.4	8.9 ± 1.5	9.1 ± 1.3*
LPA (min∙day)	298.0 ± 49.7	299.0 ± 51.9	297.0 ± 46.3
MVPA (min∙day)	68.6 ± 26.2	70.8 ± 27.7	65.5 ± 23.4*

*PHV* Peak height velocity, *TMI* Tri-ponderal mass index, *WC* Waist circumference, *WHtR* Waist-to-height ratio, *SBP* Systolic blood pressure, *DBP* Diastolic blood pressure, *MBP* Mean blood pressure, *LPA* Light physical activity, *MVPA* Moderate to vigorous physical activity, *p < 0.05, **p < 0.001. Bold values indicate statistical significance

Table 2 presents the correlation matrix among anthropometric indicators, BP variables, and physical activity in children with normal weight. No significant associations were observed between anthropometric indicators and BP variables. MVPA on weekdays and MVPA total showed positive associations with SBP (p < 0.05). Additionally, TMI was positively associated with all MVPA variables (p < 0.05).

**Table 2:** Correlation matrix among anthropometric indicators, blood pressure variables, and physical activity in children with normal weight

	SBP	DBP	MBP	WC	WHtR	TMI	MVPA weekdays	MVPA weekend	MVPA total
SBP	-								
DBP	**0.58**	-							
MBP	**0.82**	0.94	-						
WC	0.06	0.007	0.03	-					
WHtR	0.03	0.03	0.03	**0.51**	-				
TMI	0.04	−0.007	0.01	**0.18**	**0.75**	-			
MVPA weekdays	**0.12**	−0.001	0.05	0.07	0.07	**0.14**	-		
MVPA weekend	0.05	−0.06	−0.02	−0.07	0.04	**0.12**	**0.50**	-	
MVPA total	**0.11**	−0.03	0.02	0.05	0.07	**0.15**	**0.92**	**0.77**	-

*SBP* Systolic blood pressure, *DBP* Diastolic blood pressure, *MBP* Mean blood pressure, *TMI* Tri-ponderal mass index, *WC* Waist circumference, *WHtR* Waist-to-height ratio, *MVPA* Moderate to vigorous physical activity, Bold values indicate statistical significance

Table 3 presents the correlation matrix among anthropometric indicators, BP variables, and physical activity in children with overweight/obesity. The data showed significant positive associations between all anthropometric indicators (WC, WHtR, TMI) and BP variables (SBP, DBP, MBP) (p < 0.05). Regarding physical activity, MVPA on weekdays, weekends, and in total was inversely associated with WC (p < 0.05). Additionally, both WHtR and TMI showed inverse associations with total MVPA (p < 0.05).

**Table 3:** Correlation matrix among anthropometric indicators, blood pressure variables, and physical activity in children with overweight/obesity

	SBP	DBP	MBP	WC	WHtR	TMI	MVPA weekdays	MVPA weekend	MVPA total
SBP	-								
DBP	**0.62**	-							
MBP	**0.84**	**0.94**	-						
WC	**0.17**	**0.12**	**0.15**	-					
WHtR	**0.12**	**0.14**	**0.15**	**0.82**	-				
TMI	**0.14**	**0.13**	**0.15**	**0.62**	**0.87**	-			
MVPA weekdays	0.04	0.08	0.08	**−0.18**	0.11	0.12	-		
MVPA weekend	0.04	0.02	0.03	**−0.13**	−0.07	−0.09	0.006	-	
MVPA total	0.05	0.02	0.04	**−0.19**	**−0.13**	**−0.18**	−0.009	**0.76**	-

*SBP* Systolic blood pressure, *DBP* Diastolic blood pressure, *MBP* Mean blood pressure, *TMI* Tri-ponderal mass index, *WC* Waist circumference, *WHtR* Waist-to-height ratio, *MVPA* Moderate to vigorous physical activity; Bold values indicate statistical significance

Despite the lack of significant bivariate associations in some variables, mediation analysis was considered theoretically and empirically justified, particularly in children with overweight/obesity. In this subgroup, all anthropometric indicators (WC, WHtR, TMI) showed significant positive associations with BP outcomes (SBP, DBP, MBP), indicating a robust total effect. Additionally, these indicators were inversely associated with MVPA, suggesting the plausibility of an indirect pathway. To test this hypothesis, we employed bootstrapping methods, which provide robust estimates without assuming normality and can detect indirect effects even in complex models.

Table 4 summarizes the findings from all tested mediation models. Evidence of partial mediation, classified as a competitive mediation, was observed only in the models where TMI was the independent variable, MVPA on weekdays or MVPA total served as mediators, and the dependent variables were DBP and MBP. In these cases, both direct and indirect effects were significant but pointed in opposite directions, suggesting a suppression or compensatory pattern in the mediation pathway. For all other combinations of anthropometric indicators (WC and WHtR), MVPA did not mediate the relationship with any BP outcome, indicating either the absence of an indirect pathway or the dominance of direct effects.

**Table 4:** Findings’ summary concerning the direct and indirect effects according to the mediation model in children with overweight/obesity

Independents variables	Mediator variables			Dependent variables
	MVPA weekdays	MVPA weekend	MVPA total	
WC	No effect	No effect	No effect	SBP
WHtR	No effect	No effect	No effect	
TMI	Direct only	Direct only	Direct only	
WC	Direct only	No effect	Direct only	DPB
WHtR	Direct only	No effect	Direct only	
TMI	Competitive	Direct only	Competitive	
WC	Direct only	No effect	Direct only	MBP
WHtR	Direct only	Direct only	Direct only	
TMI	Competitive	No effect	Competitive	

*WC* Waist circumference, *WHtR* Waist-to-height ratio, *TMI* Tri-ponderal mass index, *MVPA* Moderate to vigorous physical activity, *SBP* Systolic blood pressure, *DBP* Diastolic blood pressure, *MBP* Mean blood pressure. All models were adjusted for sex, PHV, height, and sedentary behavior

**Fig. 2:**
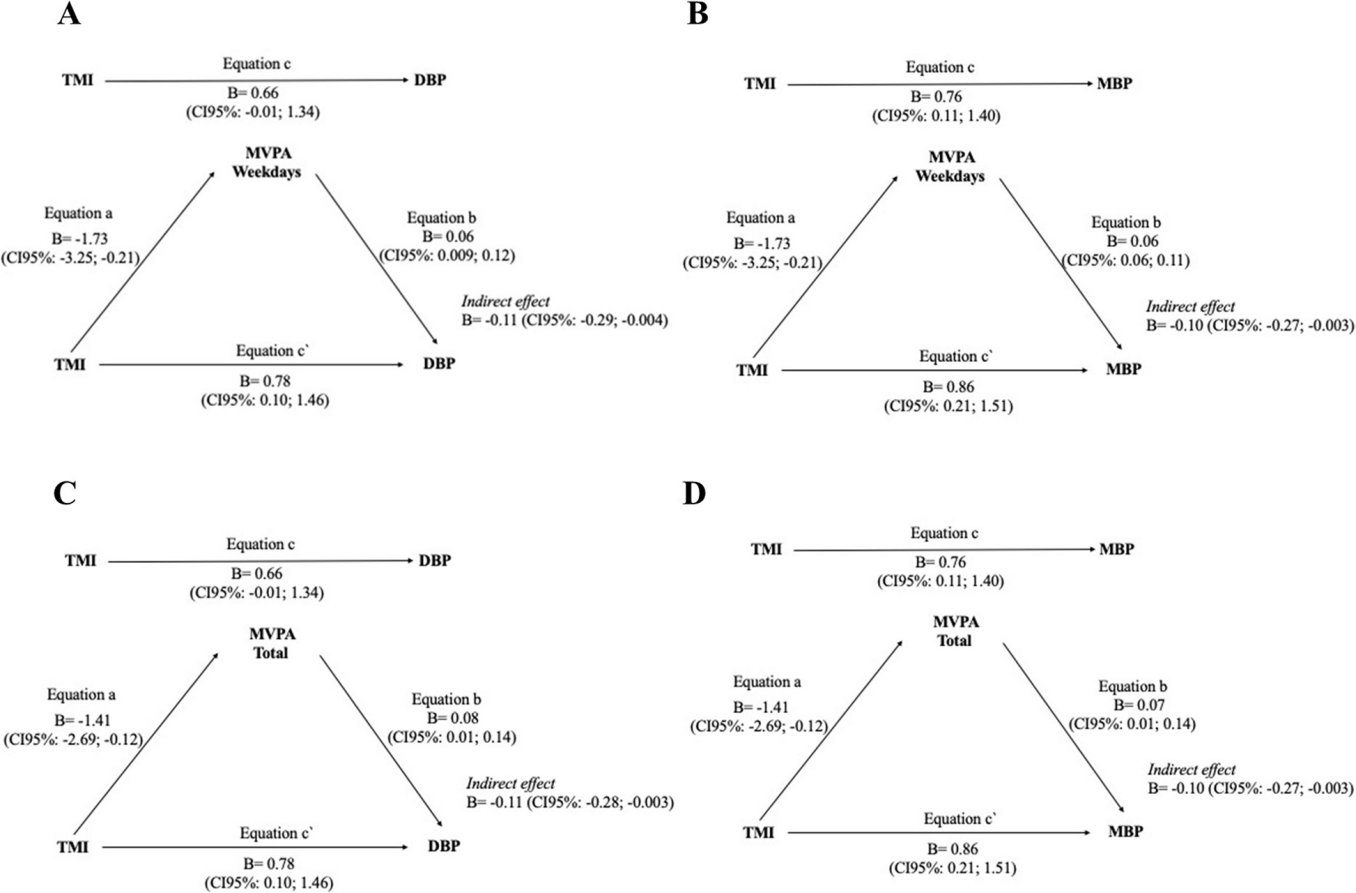
Mediation models showing the indirect and direct effects of TMI on DBP and MBP through MVPA on weekdays (A and B) and total MVPA (C and D), in children with overweight/obesity. DBP. Diastolic blood pressure; MBP. Mean blood pressure; TMI. Tri-ponderal mass index; MVPA. Moderate to vigorous physical activity. All models were adjusted for sex, PHV, height, and sedentary behavior

For the cases in which competitive mediation was identified, Fig. 2 provides a more detailed representation of the mediation models, including estimates of the total, direct, and indirect effects. The analysis revealed a significant indirect effect of TMI on DBP through MVPA on weekdays (b = −0.11, 95% CI −0.29 to −0.004), accompanied by a significant positive direct effect (b = 0.78, 95% CI 0.10 to 1.46), consistent with a competitive mediation pattern. A similar result was observed for MBP. This pattern was also replicated when MVPA total was included as the mediator.

## Discussion

The present study provides evidence that physical activity functions as a competitive mediator in the relationship between TMI and BP in children with overweight/obesity, revealing a compensatory mechanism with significant implications for pediatric cardiovascular health. Our findings indicate that the significant positive direct effect demonstrates that higher TMI directly elevates DBP and MBP through well-established pathophysiological mechanisms, including enhanced sympathetic nervous system activity, insulin resistance, and altered vascular function. Concurrently, the significant negative indirect effect indicates that increased adiposity reduces MVPA weekday and MVPA total engagement, which in turn influences BP regulation.

This competitive pattern suggests that the direct adiposity pathway predominates over any protective effects of MVPA on children with overweight/obesity. Although statistically significant, the indirect effects identified were small (β = − 0.11), indicating limited physiological impact. Comparable findings were reported by Lucena Filho et al. [18], who also observed minor indirect pathways between physical activity and DBP.

We observed that the differences in BP levels between children with normal weight and children with overweight/obesity were relatively small. This can be explained by the fact that most of the sample in the study was in the prepubertal stage, when hemodynamic changes related to excess adiposity are still subtle and less clinically evident. Previous studies indicated that more evident differences in BP arise during adolescence, when somatic maturation exerts a stronger influence on vascular function and hemodynamic regulation [13, 40]. The absence of associations in normal weight children may be related to the limited differentiation of fat distribution before puberty, reducing the likelihood that adiposity alone explains BP variability in this age group.

Our findings reveal a novel competitive mediation pattern that fundamentally differs from traditional conceptualizations of the adiposity—physical activity—BP relationship. While extensive research has established independent associations between excess adiposity and elevated BP [13, 28, 40], as well as the beneficial effects of physical activity in improving cardiovascular health and reducing BP levels [2, 3, 35, 36], the present study demonstrates that the direct adiposity pathway predominates over any protective effects of physical activity.

The competitive mediation pattern identified in our study, where the direct adiposity pathway predominates over physical activity-mediated effects, represents an important nuance extending current evidence in pediatric cardiovascular research. While conventional studies have consistently demonstrated independent beneficial effects of physical activity on BP reduction in children [2, 3, 35, 36] our findings reveal that these protective effects are substantially attenuated when competing against direct adiposity-mediated pathways. This contrasts with the mediation framework proposed by Lucena Filho et al. [18], who demonstrated that WC and skinfolds partially mediated the physical activity-BP relationship, suggesting that activity's cardiovascular benefits operate primarily through adiposity reduction.

However, the predominance of the direct effect may be conceptually consistent with previously proposed threshold phenomena documented in large pediatric cohorts, where cardiovascular benefits of lifestyle interventions become apparent only below specific adiposity cut points [19]. Biologically, excess adiposity may impair endothelial nitric oxide bioavailability, increase vascular resistance and promote DBP elevation. Simultaneously, reduced MVPA limits shear-stress-mediated vascular adaptation, reinforcing this hemodynamic imbalance.

The differential mediation patterns between weekday and weekend MVPA, and the specificity for DBP rather than SBP, reveal distinct physiological mechanisms underlying the competitive mediation framework observed in our study. The significant mediation effect demonstrated exclusively through weekday MVPA (indirect effect: b = −0.10, 95% CI −0.27; −0.003) indicates that structured daily activity patterns are more sensitive to adiposity-related reductions, subsequently contributing to elevated BP through decreased engagement in regular physical activity. This temporal specificity reflects the fact that DBP regulation, which primarily involves peripheral vascular resistance and autonomic nervous system function, is more susceptible to disruptions in consistent movement patterns compared to sporadic weekend activities [8, 26].

In contrast, the absence of significant mediation effects for SBP suggests that systolic pressure regulation, which is more strongly influenced by cardiac contractility and arterial stiffness, may be less sensitive to activity-related fluctuations in this pediatric population with overweight/obesity [31, 32].

Among the different mediation models tested in the present study using TMI, WC, and WHtR, only the model involving TMI revealed a significant mediating effect of physical activity on BP outcomes. This finding may reflect both the physiological sensitivity of TMI to overall adiposity and its methodological advantages in pediatric populations. TMI has been shown to account for better variations in body composition during growth by incorporating height in a cubic relationship, making it less prone to misclassification compared to BMI or linear indices such as WC and WHtR [27, 37]. Furthermore, TMI appears to be more strongly associated with markers of cardiometabolic risk, including BP, lipid profile, and insulin resistance, than central adiposity indicators in some pediatric samples [2, 3, 33].

This enhanced sensitivity may explain why the competitive mediation pattern, where adiposity simultaneously elevates BP directly while reducing protective physical activity engagement, emerged only when using TMI as the exposure variable, suggesting that more precise adiposity measurement is necessary to detect these complex mediational relationships in pediatric populations with excess weight. The absence of mediation for WC or WHtR reinforces that central adiposity alone may not sufficiently explain BP variability in this age group, possibly due to the limited differentiation of fat distribution before puberty.

Importantly, our findings reveal that this mediation relationship operates exclusively in children with overweight/obesity, with no significant associations observed in children with normal weight. This suggests that adiposity reaches a threshold where it begins to meaningfully influence both peripheral vascular function and structured movement behaviors. Similar thresholds have been documented in the literature. For instance, only children in the highest adiposity tertile exhibited a positive association between MVPA and both SBP and DBP [18]. Likewise, in a large prospective cohort, the relationship between childhood adiposity and hypertension relationships become pronounced only above specific BMI percentiles, with stronger effects observed for diastolic components compared to systolic components [19].

The present study presents some strengths that enhance its contribution to the pediatric cardiovascular literature. Our investigation specifically focused on children aged 6–10 years, a critical developmental period that has received limited attention in studies evaluating BP measures. We employed a comprehensive mediation framework that compared multiple anthropometric indicators (TMI, WC, and WHtR) across different temporal physical activity patterns (weekday, weekend, and total MVPA).

Additionally, our stratified analytical approach by weight status revealed that mediation mechanisms operate exclusively in children with overweight/obesity, providing clinically relevant insights that acknowledge differential physiological pathways across weight categories within this younger cohort and highlighting the importance of targeted approaches for different pediatric populations. Finally, unlike prior investigations that relied on self-reported activity logs, the use of wrist-worn accelerometry provided objective and continuous measurement of MVPA, minimizing recall bias and improving the temporal resolution of activity patterns. This method ensured accurate and reliable assessment of both physical activity and sedentary behavior.

Several limitations warrant consideration when interpreting our findings. First, the cross-sectional design precludes establishment of causal relationships between anthropometric indicators, physical activity, and BP components. Reverse causation cannot be excluded, as higher BP may reduce children’s willingness to engage in vigorous activity. Second, our BP assessment protocol involved measurements conducted within the school environment during a single evaluation session, without employing ambulatory BP monitoring for confirmation of elevated readings. Third, although our sample consisted of children, information regarding menarche was not assessed, which may represent a potential limitation given its influence on growth, body composition, and BP regulation. Fourth, dietary intake was not considered, which represents an additional limitation given its relevance to both body composition and BP. Finally, potential confounding factors such as socioeconomic status, and lifestyle variables were not adjusted for and should be addressed in future studies. These methodological constraints require cautious interpretation of the findings and highlight the need for longitudinal designs with repeated measurements to better establish the directionality of the associations observed.

## Conclusion

In conclusion, a competitive mediation pattern was observed between MVPA and BP only when the TMI was used as an indicator of adiposity and exclusively in children with overweight/obesity, rather than across all adiposity indicators. The magnitude of the indirect effects was small, suggesting that physical activity may play a limited compensatory role against the direct impact of excess weight on BP. The competitive mediation pattern indicates that excess weight simultaneously elevates BP directly while reducing protective physical activity engagement. It is also important to note that no mediation or significant associations were found in children with normal weight, and that in this group, MVPA was positively associated with SBP, indicating that the relationship between physical activity and BP is not uniform across weight categories. These findings underscore the necessity for integrated therapeutic approaches that prioritize comprehensive weight management as the primary intervention target, complemented by structured physical activity programs to address both pathways of this competitive mediation framework and optimize cardiovascular outcomes in children with overweight/obesity.

## Data Availability

The datasets and/or analyzed during the study are available from the corresponding author on request. Requests can be sent to the e-mail mctadiotto@gmail.com.

## Abbreviations

BMI-z: Body mass index score z
BP: Blood pressure
DBP: Diastolic blood pressure
ENMO: Euclidean norm minus one
LPA: Light physical activity
MBP: Mean blood pressure
MVPA: Moderate-to-vigorous physical activity
SBP: Systolic blood pressure
TMI: Tri-ponderal mass index
WC: Waist circumference
WHtR: Waist-to-height ratio

## References

[1] Ashwell M, Gunn P, Gibson S. Waist-to-height ratio is a better screening tool than waist circumference and BMI for adult cardiometabolic risk factors: systematic review and meta-analysis. Obes Rev. 2012;13(3):275–86. 22106927 10.1111/j.1467-789X.2011.00952.x

[2] Chen J, Wang Y, Li W, et al. Physical activity and eating behaviors patterns associated with high blood pressure among Chinese children and adolescents. BMC Public Health. 2023;23(1):1516. 37558994 10.1186/s12889-023-16331-1 PMC10413547

[3] Chen R, Ji L, Ma L, et al. Accuracy and capability of tri-ponderal mass index in assessing cardio-metabolic risk factors in Chinese children and adolescents aged 3 to 17 years, compared with body mass index. Chin Med J. 2023;136(11):1339–48. 36848203 10.1097/CM9.0000000000002349 PMC10309518

[4] de Onis M, Onyango AW, Borghi E, Siyam A, Nishida C, Siekmann J. Development of a WHO growth reference for school-aged children and adolescents. Bull World Health Organ. 2007;85(9):660–7. 18026621 10.2471/BLT.07.043497 PMC2636412

[5] Falkner B, Gidding SS, Baker-Smith CM, et al. Pediatric primary hypertension: an underrecognized condition: a scientific statement from the American Heart Association. Hypertension. 2023;80(6):101–11. 10.1161/HYP.0000000000000228 36994715

[6] Field A. Discovering statistics using IBM SPSS statistics: 4th edition. London: Sage; 2013.

[7] Fontes PADS, Siqueira JH, Martins HX, et al. Sedentary behavior, dietary habits, and cardiometabolic risk in physically active children and adolescents. Arq Bras Cardiol. 2023;120(2):e20220357. 36753072 10.36660/abc.20220357 PMC9882420

[8] Green DJ, Maiorana A, O’Driscoll G, Taylor R. Effect of exercise training on endothelium-derived nitric oxide function in humans. J Physiol. 2004;561(1):1–25. 15375191 10.1113/jphysiol.2004.068197 PMC1665322

[9] Hayes AF. Introduction to mediation, moderation, and conditional process analysis: a regression-based approach. The Guilford Press, 2022.

[10] Hildebrand M, Hansen BH, van Hees VT, Ekelund U. Evaluation of raw acceleration sedentary thresholds in children and adults. Scand J Med Sci Sports. 2017;27(12):1814–23. 27878845 10.1111/sms.12795

[11] Hildebrand M, Van Hees VT, Hansen BH, Ekelund U. Age Group comparability of raw accelerometer output from wrist- and hip-worn monitors. Med Sci Sports Exerc. 2014;46(9):1816–24. 24887173 10.1249/MSS.0000000000000289

[12] Hu J, Zhong Y, Ge W, et al. Comparisons of tri-ponderal mass index and body mass index in discriminating hypertension at three separate visits in adolescents: a retrospective cohort study. Front Nutr. 2022;9:1028861. 36324625 10.3389/fnut.2022.1028861 PMC9618711

[13] Huang RC, Burrows S, Mori TA, Oddy WH, Beilin LJ. Lifecourse adiposity and blood pressure between birth and 17 years old. Am J Hypertens. 2015;28(8):1056–63. 25600223 10.1093/ajh/hpu266

[14] Hunt ET, Brazendale K, De Moraes ACF, et al. Physical activity and sedentary time among U.S. adolescents before and during COVID-19: findings from a large cohort study. AJPM Focus. 2024;3(5):100253. 39175501 10.1016/j.focus.2024.100253 PMC11340494

[15] Kuciene R, Dulskiene V. Associations between tri ponderal mass index, body mass index, and high blood pressure among children and adolescents: a cross sectional study. Sci Rep. 2023;13:18148. 37875577 10.1038/s41598-023-45432-5 PMC10598122

[16] Leite N, Tadiotto MC, Menezes-Junior FJ, et al. Reduction in blood pressure and metabolic profile in overweight hypertensive boys participating in a 12-week aerobic exercise program. Eur J Pediatrics. 2024;183:4659–70. 10.1007/s00431-024-05734-w 39177754

[17] Lopes C, Torres D, Oliveira A, et al. Inquérito Alimentar Nacional e de Atividade Física, IAN-AF 2015–2016: Relatório de Resultados. Universidade do Porto; 2017.

[18] Lucena Filho A, Lima RA, Soares FC, Bezerra J, de Barros MVG. The role of adiposity in the association between physical activity and blood pressure in children. Res Q Exerc Sport. 2022;93(3):578–84. 34653344 10.1080/02701367.2021.1878089

[19] Ma S, Liu X, Lin R, et al. Childhood body size, adulthood adiposity, underlying mechanisms, and risk of incident hypertension: a prospective cohort study of 180,527 participants. BMC Med. 2025;23(1):47. 39871294 10.1186/s12916-025-03884-8 PMC11773732

[20] Malavazos AE, Capitanio G, Milani V, et al. Tri-ponderal mass index vs body mass index in discriminating central obesity and hypertension in adolescents with overweight. Nutr Metab Cardiovasc Dis. 2021;31:1613. 33741212 10.1016/j.numecd.2021.02.013

[21] Martins J, Augusto C, Silva MJ, et al. Effectiveness of a health promotion program on overweight in vulnerable children from primary schools (BeE-school): a cluster-randomized controlled trial. Int J Obes. 2025;49(2):332. 10.1038/s41366-024-01672-7 39521923

[22] Mirzaei M, Taylor R, Morrell S, Leeder SR. Predictors of blood pressure in a cohort of school-aged children. Eur J Cardiovasc Prev Rehabil. 2007;14(5):624–9. 17925620 10.1097/HJR.0b013e32828621c6

[23] Moore SA, McKay HA, MacDonald H, et al. Enhancing a somatic maturity prediction model. Med Sci Sports Exerc. 2015;47(8):1755–64. 25423445 10.1249/MSS.0000000000000588

[24] Nimkarn N, Sewarit A, Pirojsakul K, et al. Waist-to-height-ratio is associated with sustained hypertension in children and adolescents with high office blood pressure. Front Cardiovasc Med. 2023;9:1026606. 36712271 10.3389/fcvm.2022.1026606 PMC9874100

[25] Nitzl C, Roldán JL, Cepeda G. Mediation analysis in partial least squares path modeling: helping researchers discuss more sophisticated models. Industrial Management & Data Systems. 2016;116(9):1849–64.

[26] Pescatello LS, Franklin BA, Fagard R, et al. Exercise and hypertension. Med Sci Sports Exerc. 2004;36(3):533. 15076798 10.1249/01.mss.0000115224.88514.3a

[27] Peterson CM, Su H, Thomas DM, et al. Tri-ponderal mass index vs body mass index in estimating body fat during adolescence. JAMA Pediatr. 2017;171(7):629. 28505241 10.1001/jamapediatrics.2017.0460 PMC5710345

[28] Pinheiro G, Mello J, Gaya A, Gaya AR. Blood pressure in children: association with anthropometric indicators, body composition, cardiorespiratory fitness and physical activity. Arq Bras Cardiol. 2021;116(5):950–6. 34008820 10.36660/abc.20190520 PMC8121480

[29] Preacher KJ, Hayes AF. Asymptotic and resampling strategies for assessing and comparing indirect effects in multiple mediator models. Behav Res Methods. 2008;40(3):879–91. 18697684 10.3758/brm.40.3.879

[30] Rodrigues PRM, Pereira RA, Gama A, et al. Body adiposity is associated with risk of high blood pressure in Portuguese schoolchildren. Rev Port Cardiol. 2018;37(4):285–92. 29685850 10.1016/j.repc.2017.09.016

[31] Safar ME, Levy BI, Struijker-Boudier H. Current perspectives on arterial stiffness and pulse pressure in hypertension and cardiovascular diseases. Circulation. 2003;107(22):2864–9. 12796414 10.1161/01.CIR.0000069826.36125.B4

[32] Sequi-Dominguez I, Mavridis D, Cavero-Redondo I, et al. Comparative effectiveness of different types of exercise in reducing arterial stiffness in children and adolescents: a systematic review and network meta-analysis. Br J Sports Med. 2023;57(15):997–1002. 36963807 10.1136/bjsports-2022-106285

[33] Shim YS. The relationship between tri-ponderal mass index and metabolic syndrome and its components in youth aged 10–20 years. Sci Rep. 2019;9(1):14462. 31594996 10.1038/s41598-019-50987-3 PMC6783432

[34] Silveira JFDC, López-Gil JF, Reuter CP, et al. Mediation of obesity-related variables in the association between physical fitness and cardiometabolic risk in children and adolescents: a systematic review and meta-analysis. BMJ Open Sport Exerc Med. 2025;11(2):e002366. 40191840 10.1136/bmjsem-2024-002366 PMC11969607

[35] Tozo JVA, Tadiotto MC, Tozo TAA, et al. Effects of different physical exercise programs on blood pressure in overweight children and adolescents: systematic review and meta-analysis. BMC Pediatr. 2025;25(1):252. 40155857 10.1186/s12887-025-05575-y PMC11951679

[36] Tozo TAA, Pereira BO, Menezes-Junior FJ, et al. Hypertensive measures in schoolchildren: Risk of central obesity and protective effect of moderate-to-vigorous physical activity. Arq Bras Cardiol. 2020;115(1):42–9. 32785497 10.36660/abc.20180391 PMC8384320

[37] Wang X, Dong B, Ma J, Song Y, Zou Z, Arnold L. Role of tri-ponderal mass index in cardio-metabolic risk assessment in children and adolescents: compared with body mass index. Int J Obes. 2020;44(4):886–94. 10.1038/s41366-019-0416-y 31332274

[38] Wiggers E, Costa GP, Ribeiro EHC, et al. Effects of school-based interventions on blood pressure in obese children: metanalysis. Rev Bras Ativ Fis Saude. 2024;29:e0330.

[39] World Health Organization. Obesity and overweight. Geneva: World Health Organization, 2025. Available from: https://www.who.int/news-room/fact-sheets/detail/obesity-and-overweight

[40] Yuan WL, Kramer MS, Michael N, et al. Trajectories of systolic blood pressure in children: risk factors and cardiometabolic correlates. J Pediatrics. 2021;236:86–94. 10.1016/j.jpeds.2021.05.027 PMC7611585 34019883

[41] Zhao X, Lynch JG, Chen Q. Reconsidering Baron and Kenny: myths and truths about mediation analysis. J Consum Res. 2010;37:197–206.

